# Effect of common genetic variants on the risk of cirrhosis in non‐alcoholic fatty liver disease during 20 years of follow‐up

**DOI:** 10.1111/liv.15438

**Published:** 2022-10-11

**Authors:** Magnus Holmer, Mattias Ekstedt, Patrik Nasr, Robin Zenlander, Axel Wester, Federica Tavaglione, Stefano Romeo, Stergios Kechagias, Per Stål, Hannes Hagström

**Affiliations:** ^1^ Division of Liver and Pancreatic disease, Department of Upper GI Karolinska University Hospital Stockholm Sweden; ^2^ Department of Medicine, Huddinge Karolinska Institutet Stockholm Sweden; ^3^ Department of Gastroenterology and Hepatology, Department of Health, Medicine, and Caring Sciences Linköping University Linköping Sweden; ^4^ Department of Molecular and Clinical Medicine, Institute of Medicine, Sahlgrenska Academy, Wallenberg Laboratory University of Gothenburg Gothenburg Sweden; ^5^ Department of Cardiology Sahlgrenska University Hospital Gothenburg Sweden; ^6^ Clinical Epidemiology Unit, Department of Medicine, Solna Karolinska Institutet Stockholm Sweden

**Keywords:** NAFLD, NASH, PNPLA3, TM6SF2

## Abstract

**Background and Aims:**

Several genotypes associate with a worse histopathological profile in patients with non‐alcoholic fatty liver disease (NAFLD). Whether genotypes impact long‐term outcomes is unclear. We investigated the importance of *PNPLA3*, *TM6SF2*, *MBOAT7* and *GCKR* genotype for the development of severe outcomes in NAFLD.

**Method:**

DNA samples were collected from 546 patients with NAFLD. Advanced fibrosis was diagnosed by liver biopsy or elastography. Non‐alcoholic steatohepatitis (NASH) was histologically defined. Additionally, 5396 controls matched for age, sex and municipality were identified from population‐based registers. Events of severe liver disease and all‐cause mortality were collected from national registries. Hazard ratios (HRs) adjusted for age, sex, body mass index and type 2 diabetes were estimated with Cox regression.

**Results:**

In NAFLD, the G/G genotype of *PNPLA3* was associated with a higher prevalence of NASH at baseline (odds ratio [OR] 3.67, 95% CI = 1.66–8.08), but not with advanced fibrosis (OR 1.81, 95% CI = 0.79–4.14). After up to 40 years of follow‐up, the *PNPLA3* G/G genotype was associated with a higher rate of severe liver disease (adjusted hazard ratio [aHR] 2.27, 95% CI = 1.15–4.47) compared with the C/C variant. NAFLD patients developed cirrhosis at a higher rate than controls (aHR 9.00, 95% CI = 6.85–11.83). The *PNPLA3* G/G genotype accentuated this rate (aHR 23.32, 95% = CI 9.14–59.47). Overall mortality was not affected by any genetic variant.

**Conclusion:**

The *PNPLA3* G/G genotype is associated with an increased rate of cirrhosis in NAFLD. Our results suggest that assessment of the *PNPLA3* genotype is of clinical relevance in patients with NAFLD to individualize monitoring and therapeutic strategies.

**Abbreviations**
BMI: body mass index
CVD: cardiovascular disease
DNA: deoxyribonucleic acid
EDTA: ethylenediaminetetraacetic acid
FFPE: formalin‐fixed paraffin‐embedded
GCKR: the glucokinase regulator gene
HCC: hepatocellular carcinoma
HR: hazard ratio
ICD: international classification of diseases
IQR: interquartile range
MBOAT7: the membrane‐bound O‐acyltransferase domain containing 7 gene
NAFLD: non‐alcholic fatty liver disease
NASH: non‐alcoholic steatohepatitis
OR: odds ratio
PCR: polymerase chain reaction
PNPLA3: the patatin‐like phospholipase domain containing 3 gene
PRS: polygenic risk score
RNA: ribonucleic acid
T2D: type 2 diabetes mellitus
TM6SF2: the transmembrane 6 superfamily member 2 gene

**Lay summary**

Studies show that certain common genetic variants are linked to an increased risk of inflammation and scaring of the liver (*fibrosis*) in people who have non‐alcoholic fatty liver disease (NAFLD). In this study, we gathered information about four genetic variants in a group of 546 persons with NAFLD, matched to up to 10 individuals from the general Swedish population. Information about the event that someone developed cirrhosis, cardiovascular disease, or died, was collected from national patient registers and we were able to follow the group over a period of 20 years. We found that NAFLD patients with a specific variant of a gene called *PNPLA3* had a higher risk of developing cirrhosis. The risk was increased both compared with that of NAFLD patients with the normal gene variant and compared with the general population.

## 1 INTRODUCTION

Non‐alcoholic fatty liver disease (NAFLD) is the most common chronic liver disease globally with an estimated prevalence of 25%.^1^ The increased prevalence of NAFLD is linked to a similar increase in obesity and type 2 diabetes mellitus (T2D).^2^, ^3^ The histopathological spectrum of NAFLD ranges from simple steatosis to non‐alcoholic steatohepatitis (NASH), fibrosis and cirrhosis.^4^ There is evidence that genetic factors significantly affect the risk of such disease progression in NAFLD.^5^, ^6^, ^7^

A large body of evidence supports associations for several genetic variants with different traits of NAFLD, including histologically defined NASH, fibrosis and cirrhosis.^8^, ^9^, ^10^, ^11^, ^12^, ^13^, ^14^, ^15^, ^16^, ^17^, ^18^, ^19^, ^20^ Some of the most studied genetic variants include the I148M variant of the patatin‐like phospholipase domain containing 3 (*PNPLA3 rs738409*) gene, the E167K variant of the transmembrane 6 superfamily member 2 (*TM6SF2 rs58542926*) gene, the membrane‐bound O‐acyltransferase domain containing 7 (*MBOAT7 rs641738*) gene and the glucokinase regulator (*GCKR rs1260326*) gene. Furthermore, since the initial discovery of an association between these four genes and NAFLD severity, several studies have investigated their combined effect on the risk of progressive disease in NAFLD. In a study on 1515 patients with histologically determined NAFLD, a polygenic risk score (PRS) was constructed. This PRS correlated to the presence of hepatic fat content, NASH and liver fibrosis.^21^

However, most of the evidence stem from studies with a cross‐sectional design and previous longitudinal cohort studies did not include patients with histologically defined NAFLD or had short time of follow‐up.^22^, ^23^, ^24^ In the present study, we performed a longitudinal multicentre study to investigate how genetic variants of *PNPLA3*, *TM6SF2*, *MBOAT7* and *GCKR* affect the risk of developing severe liver disease, cardiovascular disease (CVD) and overall mortality in a cohort of well‐characterized patients with NAFLD with extensive follow‐up.

## 2 MATERIALS AND METHODS

### 2.1 Study population

#### 2.1.1 The NAFLD population

**FIGURE 1:**
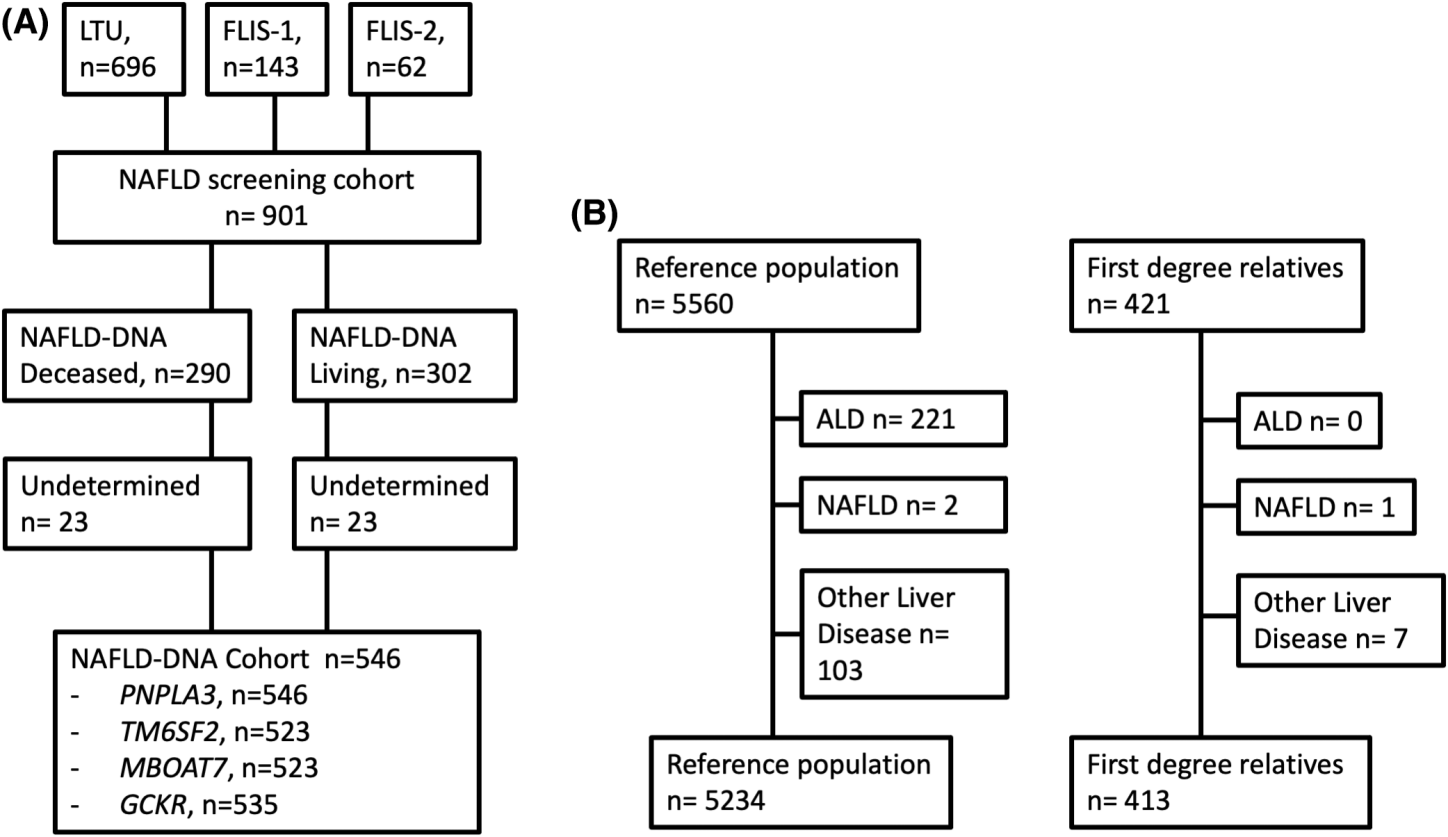
Flowchart describing patient selection and exclusion criteria in the NAFLD cohorts (A) and matched reference population and first‐degree relatives (B). Abbreviations: LTU = the Long‐term follow‐up of NAFLD study. FLIS‐1 and 2 = the Fatty Liver in Sweden Study 1 and 2, NAFLD, non‐alcoholic fatty liver disease, ALD , alcohol‐related liver disease.

A total of 901 individuals diagnosed with NAFLD at the Karolinska University Hospital or Linköping University Hospital, both Sweden, between 1974 and 2019 were screened for inclusion. Subjects were collected from an ongoing study, Fatty Liver In Sweden 2 (FLIS‐2, *n* = 62) and from two previously described study cohorts, Fatty liver in Sweden 1 (FLIS‐1, *n* = 143) and long‐term follow‐up of NAFLD (LTU, *n* = 696).^25^, ^26^ The NAFLD diagnosis was established by standard clinical investigation, that is, confirmed steatosis on imaging or liver biopsy in combination with alcohol consumption of less than 140 g/week for women and 210 g/week for men and the exclusion of any other chronic liver disease or steatogenic medications. Patients with concurrent liver diseases such as alcohol‐related liver disease, chronic viral hepatitis B and C were excluded. Samples for DNA analysis could be collected from 592 NAFLD subjects, 302 from living and 290 from deceased subjects. In total, 546 samples were successfully characterized for PNPLA3 allele type, 523 for *TM6SF2*, 532 for *MBOAT7* and 535 for *GCKR*. Figure 1A describes the inclusion of study participants. Blood tests, anthropometric measures and data on comorbidities were collected at baseline.

#### 2.1.2 Reference population and first‐degree relatives

For each subject with NAFLD, up to 10 reference individuals from the Swedish general population, matched for age, sex and municipality at the year of diagnosis were identified by linkage to the Total Population Register.^27^ This matched cohort was subsequently linked to several other national registers (described below), allowing for identification of outcomes and registry‐based covariates. After exclusion of reference individuals with a diagnosis of alcohol use disorders (*n* = 221), other chronic liver diseases (n = 105) or NAFLD before baseline (*n* = 2), the reference population consisted of 5234 individuals. A total of 421 first‐degree siblings of the NAFLD patients were also identified in the Total Population Register. Of these, one was excluded due to a diagnosis of NAFLD before baseline and seven due to other liver diseases. Thus, 413 siblings were included in the analysis (Figure 1B).

### 2.2 Histological assessment

#### 2.2.1 NASH and liver fibrosis

Liver biopsy had been performed in 496 subjects (90.8%). The NAFLD activity score (NAS) was calculated by summing the degree of steatosis (0–3), lobular inflammation (0–3) and hepatocellular ballooning (0–2) according to Kleiner et al.^28^ The fatty liver inhibition of progression algorithm was used to define the presence of NASH.^29^ Fibrosis stage was assessed according to the classification by Kleiner.^28^ In subjects where liver biopsy was not available transient elastography (Fibroscan® Echosens) was used to categorize fibrosis. A liver stiffness of ≥15 kPa was defined as advanced fibrosis.

### 2.3 Genetic analysis

#### 2.3.1 Collection of DNA samples

Subjects in the original NAFLD cohorts that were alive at the time of inclusion (2017–2019) were contacted by telephone or mail and asked to leave a blood sample for DNA analysis at the outpatient clinic at the Department of Hepatology, Karolinska University Hospital. Subjects who were not able to come to the clinic were asked to leave a blood test at a primary care laboratory, which was subsequently shipped to the Karolinska University Hospital for analysis. For individuals who were deceased, tissue for DNA analysis was extracted from historical liver biopsies stored in the medical biobanks at each hospital.

#### 2.3.2 DNA isolation and genotyping

DNA was extracted from whole blood in EDTA tubes using the QiaSymphony SP instrument (Qiagen) and the DNA Mini Kit (Qiagen, Cat. No. 937236) according to the manufacturer's manual. In short, erythrocytes and leucocytes were lysed, and free DNA was bound to paramagnetic silica particles. The particles were extracted using magnets, and finally the DNA was eluated.

DNA from formaldehyde‐fixed, paraffin‐embedded (FFPE) liver biopsies was performed using RecoverAll™ Multi‐Sample RNA/DNA Isolation Workflow (Invitrogen, Massachusetts, USA Cat#A26069). Three 10‐μm sections of FFPE blocks were processed according to the manufacturer's protocol (User bulletin, Version: MAN0010642 Rev. D.0) and as previously described.^30^ Briefly, the FFPE slides were deparaffinized and digested with protease. The samples were then loaded on pure link columns. The DNA bounded to the column was eluted in a fresh tube while the flow‐through RNA containing was applied to a new column, treated with DNase, and then eluted from the filter to recover RNA.

Genotyping was performed using TaqMan probes.^31^ The genotypes of *PNPLA3* (rs738409), *TM6SF2* (rs58542926), *MBOAT7* (rs641738) and *GCKR* (rs1260326) were analysed using commercially available TaqMan probes and a 7500 Fast Real‐Time PCR System, both from Thermo Fisher, according to the manufacturer's instruction.^30^

#### 2.3.3 Calculation of genetic risk score

We used the model previously described by Dongiovanni et al. to calculate a PRS.^21^ The score summarizes the total number of alleles in the four investigated genotypes weighted by their individual effect size using linear regression and was shown to correlate strongly to histopathological traits of NASH and fibrosis stage.^21^

### 2.4 Outcomes

#### 2.4.1 Severe liver disease, cardiovascular events and overall mortality

Every individual residing in Sweden has a unique personal identity number that is linked to several registers, including the National Patient Register,^32^, ^33^ the Swedish Cancer Register^34^ and the Causes of Death Register.^35^ For both the NAFLD cohort and the reference population, we received data from these registers on diagnoses of other liver diseases than NAFLD, cirrhosis, decompensation events, liver transplantation, cardiovascular events, alcohol‐ and drug‐associated disorders, hepatocellular carcinoma (HCC) and date and cause of death. All subjects were followed from the date of inclusion until the date of an outcome, a censoring event, or 31 December 2019. Censoring events in the analysis of overall mortality were emigration, liver transplantation or diagnosis of liver diseases other than NAFLD after the index date. In the analyses of CVD and severe liver disease, death *not* due to the outcome (CVD‐ or liver‐related, respectively) were also considered a censoring event. The ICD‐8, ICD‐9 and ICD‐10 systems were used to define outcomes in the registries. Cardiovascular events were defined as acute ischaemic heart disease or acute cerebrovascular disease. Severe liver disease was defined as a diagnosis of cirrhosis, decompensation with ascites, oesophageal varices, hepatic encephalopathy, portal hypertension, hepatorenal syndrome or HCC. The ICD codes used to define outcomes are shown in Table S1.

### 2.5 Statistical analysis

Baseline characteristics of the NAFLD cohort were calculated using summary statistics and are shown as median values with interquartile ranges (IQR) for continuous parameters or as total numbers and percentages for categorical parameters. The association between allele type and the presence of NASH and advanced fibrosis (stage 0–2 vs stage 3–4, or less or more than 15 kPa in those with only Fibroscan assessment of fibrosis) at baseline was calculated only in patients with NAFLD (as there were no genetic data in reference individuals or siblings), using logistic regression presented as odds ratios (OR) with 95% confidence intervals (CI). In a second model, we adjusted for age, sex, body mass index (BMI) and T2D at baseline.

Cox regression was used to estimate rates of severe liver disease, cardiovascular events and overall mortality respectively. In the NAFLD population, we compared subgroups of patients with NAFLD per allele type, using the wild type as the reference group. The primary regression model was unadjusted, whereas the second model was adjusted for age, sex, T2D and BMI. As fibrosis might not be a confounder, but rather a mediator, we did not adjust for fibrosis in the model. Instead, we stratified the cohort on the presence of advanced fibrosis at baseline and examined the rates of each outcome separately in these strata. However, as a sensitivity analysis, we additionally adjusted the regression model for advanced fibrosis.

Separately, we investigated rates of outcomes in NAFLD compared with matched controls per allele type (e.g. comparing patients with *PNPLA3* G/G to matched controls and *PNPLA3* C/C to matched controls, separately). This Cox model was conditioned on the matching factors (age, sex, calendar year of diagnosis and municipality). Estimates from the Cox models are presented as hazard ratios (HRs) with 95%CIs. The aim of the study was to examine the etiological association between genotypes and outcomes. Therefore, we did not use a competing risk framework as the main analysis. Cox regression is preferred when the research question is considering etiological associations, whereas the competing risk framework is preferred when calculating cumulative incidence.^36^ However, as a second sensitivity analysis, we performed a competing risk analysis using the Fine‐Gray regression model where death from other causes than severe chronic liver disease was defined as competing risk.

### 2.6 Ethical considerations

The study was approved by the regional ethics committee in Stockholm, Sweden. All subjects included in the NAFLD cohorts that were alive signed an informed consent at the time of collection of the DNA sample (Dnr 2011/905‐31/2, 2011/13‐31/1, 2016/2137–31, 2018/880–31).

## 3 RESULTS

### 3.1 Patient selection and baseline characteristics

The median age of the NAFLD cohort was 51 years (IQR 39–59), and 340 patients (62%) were male. Median BMI was 27.4 kg/m^2^ (IQR 25.0–30.1), and 100 subjects had T2D (19.1%). Complete data on fibrosis stage from biopsy were available in 496 subjects. Advanced fibrosis (fibrosis stage 3–4) was present in 72 (14.5%) subjects. Transient elastography was used to categorize fibrosis in 34 subjects who lacked liver biopsy of which seven were classified as having advanced fibrosis (liver stiffness ≥15 kPa). Most subjects were included after 1990 (53.1%), and only 15 subjects were included before 1980. The distribution of genetic variants for each gene and complete baseline characteristics of the NAFLD cohort are shown in Table 1.

**TABLE 1:** Baseline characteristics of the NAFLD cohort included in the DNA analysis

Parameter	Complete data in *N* persons (%)	Median/IQR or *N*/%
Age (years)	546 (100)	51 (39–59)
Sex (male)	546 (100)	340 (62.2)
BMI (kg/m^2^)	483 (88)	27.4 (25.0–30.1)
Year of diagnosis	546 (100)	
<1980	—	15 (2.7)
1980–1989	—	241 (44.1)
1990–1999	—	158 (28.9)
2000–2009	—	76 (13.9)
>2009	—	56 (10.3)
ALT (IU/L)	535 (98.0)	66.4 (45.3–98.9)
*PNPLA3* (CC/CG/GG)	546 (100)	231/230/85
*TM6SF2* (CC/CT/TT)	523 (95.8)	386/116/21
*MBOAT7* (CC/CT/TT)	532 (97.4)	187/234/111
*GCKR* (CC/CT/TT)	535 (98.0)	209/219/107
Liver biopsy available	496 (90.8)	
Fibrosis stage		
0	—	122 (24.6)
1	—	192 (38.7)
2	—	110 (22.2)
3	—	51 (10.3)
4	—	21 (4.2)
Fat score	450 (82.4)	
0	—	22 (4.9)
1	—	164 (36.4)
2	—	109 (24.2)
3	—	155 (34.4)
NASH	442 (81.0)	288 (65.2)
Type 2 diabetes mellitus	524 (96.0)	100 (19.1)
Liver stiffness (kPa)	61 (11.2)	9.9 (6.8–16.0)

Abbreviations: ALT, alanine aminotransferase; BMI, body mass index; DNA, deoxyribonucleic acid; IQR, interquartile range; NAFLD, non‐alcoholic fatty liver disease; NASH, non‐alcoholic steatohepatitis.

### 3.2 Association between allele type and severity of NAFLD at baseline

In the NAFLD cohort, the G/G‐genotype of *PNPLA3* was associated with a higher risk of NASH in both crude (OR 3.42, 95% CI = 1.68–6.95) and adjusted analyses (aOR 3.67, 95% CI = 1.66–8.08), while no association with any allele type of *TM6SF2*, *MBOAT7* or *GCKR* and the presence of NASH was seen. The PRS was associated with a higher risk of NASH in both crude and adjusted analysis (OR 3.59 per unit increase, 95% CI = 1.53–8.43; aOR 3.81 95% CI = 1.48–9.81); hence, this was largely driven by the *PNPLA3* component. No association was seen between *PNPLA3*, *TM6SF2*, *MBOAT7*, *GCKR* or the PRS and advanced fibrosis (Table 2).

**TABLE 2:** Associations between genotype or PRS and NAFLD severity (NASH and advanced fibrosis) at baseline using logistic regression

Gene type	NASH				Advanced fibrosis			
	*N* exposed	*N* outcomes (%)	OR (95%CI)	aOR^a^ (95%CI)	*N* exposed	*N* outcomes (%)	OR (95%CI)	aOR^a^ (95%CI)
*PNPLA3* (C/C)	192	114 (59.4)	1.0 (ref)	1.0 (ref)	227	30 (13.2)	1.0 (ref)	1.0 (ref)
*PNPLA3* (C/G)	184	119 (64.7)	1.25 (0.83–1.90)	1.34 (0.84–2.11)	222	35 (15.8)	1.23 (0.72–2.08)	1.78 (0.93–3.39)
*PNPLA3* (G/G)	66	55 (83.3)	3.42 (1.68–6.95)	3.67 (1.66–8.08)	81	14 (17.3)	1.49 (0.76–2.94)	1.81 (0.79–4.14)
*TM6SF2* (C/C)	320	210 (65.6)	1.0 (ref)	1.0 (ref)	374	60 (16.0)	1.0 (ref)	1.0 (ref)
*TM6SF2* (C/T)	93	60 (64.5)	0.95 (0.59–1.54)	1.07 (0.63–1.83)	115	15 (13.0)	0.79 (0.43–1.44)	0.69 (0.33–1.43)
*TM6SF2* (T/T)	16	10 (62.5)	0.87 (0.31–2.46)	0.87 (0.27–2.80)	21	2 (9.5)	0.84 (0.24–2.93)	1.05 (0.22–5.10)
*GCKR* (C/C)	166	107 (64.5)	1.0 (ref)	1.0 (ref)	200	30 (15.0)	1.0 (ref)	1.0 (ref)
*GCKR* (C/T)	184	119 (64.7)	1.01 (0.65–1.56)	0.79 (0.49–1.28)	213	31 (14.5)	0.96 (0.56–1.66)	0.93 (0.49–1.79)
*GCKR* (T/T)	85	57 (67.1)	1.12 (0.65–1.95)	0.97 (0.53–1.77	106	16 (15.1)	1.01 (0.52–1.94)	1.14 (0.54–2.42)
*MBOAT7* (C/C)	157	96 (61.1)	1.0 (ref)	1.0 (ref)	180	28 (15.6)	1.0 (ref)	1.0 (ref)
*MBOAT7* (C/T)	193	125 (64.8)	1.17 (0.75–1.81)	1.26 (0.78–2.04)	229	32 (14.0)	0.88 (0.51–1.53)	0.87 (0.45–1.68)
*MBOAT7* (T/T)	80	58 (72.5)	1.67 (0.93–3.01)	1.48 (0.78–2.83)	107	16 (14.9)	0.95 (0.49–1.86)	1.27 (0.58–2.81)
PRS	419	273 (65.2)	3.59 (1.53–8.43)	3.81 (1.48–9.81)	496	78 (15.7)	0.96 (0.35–2.59)	1.56 (0.48–5.13)

*Note*: For PRS, the OR shows the association between PRS = 1 and the outcome compared with a PRS =0 (ref.).

Abbreviations: aOR, adjusted odds ratio; BMI, body mass index; CI, confidence interval; NAFLD, non‐alcoholic fatty liver disease; NASH, non‐alcoholic steatohepatitis; OR, odds ratio; PRS, polygenic risk score.

^a^ Adjusted for age, sex, BMI and type 2 diabetes.

### 3.3 Association between allele type and rate of development of severe liver disease in NAFLD

In the analysis restricted to the NAFLD cohort, over a median follow‐up of 19.6 (0.1–40.0) years, 78 events of severe liver disease were observed, of which there were 35 cases of liver decompensation and 20 cases of HCC. The G/G‐genotype of *PNPLA3* was associated with a higher rate of developing severe liver disease compared with the C/C genotype in both the crude analysis (HR 2.14, 95% CI = 1.17–3.91) and when adjusting for age, sex, T2D and BMI (aHR 2.27, 95% CI = 1.15–4.47) (Table 3).

**TABLE 3:** Hazard ratios from Cox regression analyses of associations between genotypes and development of severe liver disease in the NAFLD cohort

Genotype	*N* exposed	*N* of outcomes (%)	Incidence rate (per 1000 person years)	HR	aHR^a^
*PNPLA3* (C/C)	231	27 (11.6)	6.3 (4.2–9.2)	1.0 (ref)	1.0 (ref)
*PNPLA3* (C/G)	230	32 (13.9)	7.5 (5.2–10.6)	1.20 (0.71–2.02)	1.26 (0.70–2.28)
*PNPLA3* (G/G)	85	19 (22.3)	13.2 (8.2–21.2)	2.14 (1.17–3.91)	2.27 (1.15–4.47)
*TM6SF2* (C/C)	386	52 (13.5)	7.8 (5.9–10.2)	1.0 (ref)	1.0 (ref)
*TM6SF2* (C/T)	116	15 (12.9)	7.2 (4.3–11.9)	0.90 (0.51–1.60)	0.85 (0.44–1.63)
*TM6SF2* (T/T)	21	3 (14.3)	6.7 (2.1–20.7)	0.93 (0.29–2.98)	1.06 (0.26–4.45)
*GCKR* (C/C)	209	31 (14.8)	8.2 (5.8–11.6)	1.0 (ref)	1.0 (ref)
*GCKR* (C/T)	219	29 (13.2)	7.7 5.4–11.1)	0.86 (0.52–1.43)	0.78 (0.44–1.38)
*GCKR* (T/T)	107	14 (13.1)	7.5 (4.4–12.7)	0.94 (0.51–1.74)	0.96 (0.49–1.86)
*MBOAT7* (C/C)	187	33 (17.6)	10.4 (7.4–14.7)	1.0 (ref)	1.0 (ref)
*MBOAT7* (C/T)	234	25 (10.7)	5.8 (3.9–8.6)	0.58 (0.35–0.99)	0.55 (0.31–0.99)
*MBOAT7* (T/T)	111	14 (12.6)	7.4 (4.4–12.6)	0.74 (0.40–1.37)	0.77 (0.39–1.54)
PRS	506	69 (13.6)	—	1.78 (0.71–4.43)	1.96 (0.69–5.58)

*Note*: For PRS, the HR shows the association between PRS = 1 and the outcome compared with a PRS = 0 (ref.).

Abbreviations: aHR, adjusted hazard ratio; BMI, body mass index; CI, confidence interval; HR, hazard ratio; NAFLD, non‐alcoholic fatty liver disease; PRS, polygenic risk score.

^a^ Adjusted for age, sex, type 2 diabetes mellitus and BMI.

In a sensitivity analysis, an association between the *PNPLA3* G/G‐genotype and the risk of both HCC (HR 4.14, 95% CI = 1.43–11.94; aHR 6.46, 95% CI = 1.86–22.44) and liver decompensation (HR 2.58, 95% CI = 1.02–6.56; aHR 2.82, 95% CI = 1.08–7.40) was observed in both unadjusted analysis and adjusted for age, sex, T2D and BMI.

**FIGURE 2:**
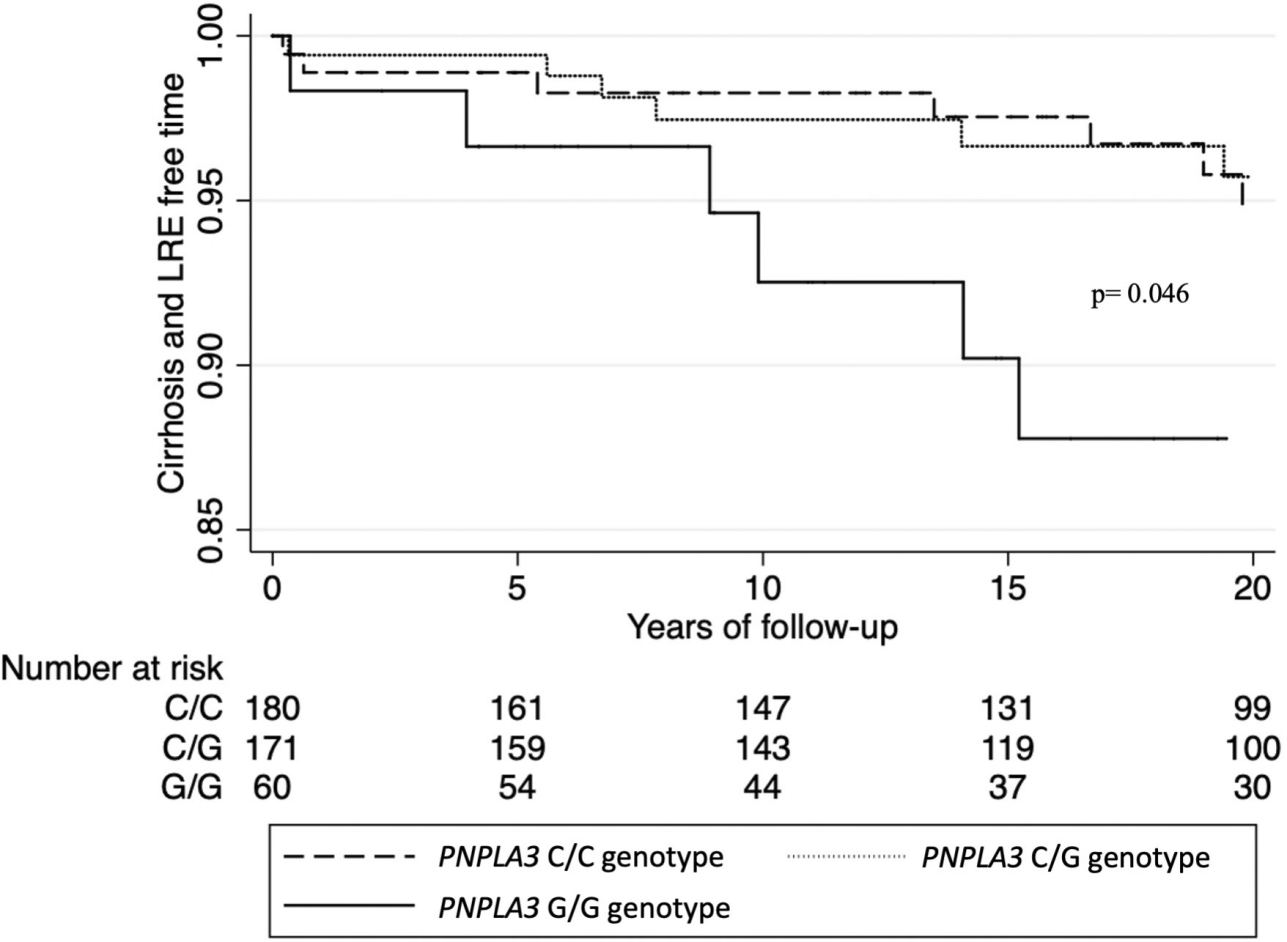
Kaplan–Meier estimate of cirrhosis‐free survival time in a subgroup of NAFLD patients without advanced fibrosis (F0‐2 or < 15 kPa). *p* = log rank test for the G/G genotype versus the C/C genotype.

When stratifying subjects on the presence of advanced fibrosis at baseline (F0‐2 or < 15 kPa, *n* = 451; F3‐4 or ≥ 15 kPa, *n* = 79) a significant association between carriage of the *PNPLA3* G/G genotype and a higher rate of development of severe liver disease was detected in the group without (aHR 2.49, 95% CI = 1.05–5.89) but not in the group with advanced fibrosis (aHR 0.89, 95% CI = 0.21–3.74) (Figure 2). In the first sensitivity analysis, which additionally included advanced fibrosis in the adjusted model, the association was no longer significant (aHR 1.68 95% CI = 0.81–3.46).

The second sensitivity analysis using the Fine‐Gray competing risk model (adjusted for age, sex, BMI and T2D, as in the Cox models) still showed a significant association between the *PNPLA3* G/G genotype and the risk of severe liver disease (sHR 2.17 95% CI = 1.19–3.95; adjusted sHR 2.21 95% CI = 1.11–4.40).

None of the polymorphisms of *TM6SF2*, *MBOAT7*, *GCKR*, nor the PRS, were associated with increased risk of development of severe liver disease in the full cohort (Table 3).

### 3.4 Association between allele type, rate of CVD and risk for overall mortality in NAFLD

**FIGURE 3:**
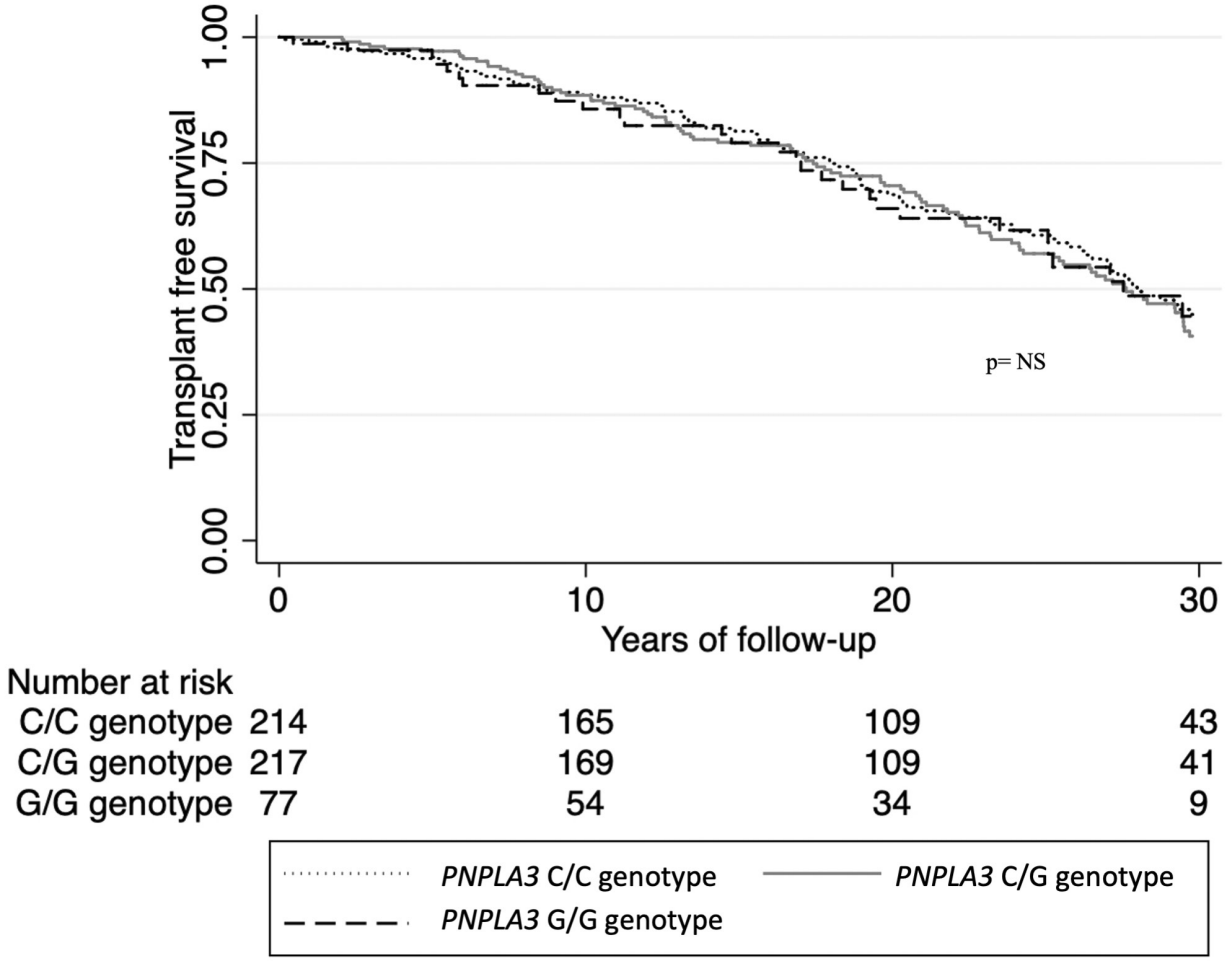
Kaplan–Meier estimate of transplant‐free survival time comparing carriers of the C/C, C/G and the G/G genotype of *PNPLA3* gene. *p* is for significance of log rank test.

A total of 195 subjects (35.7%) in the NAFLD cohort had a CVD outcome. The incidence rate of cardiovascular events did not differ significantly between any allele type of *PNPLA3*, *TM6SF2*, *MBOAT7* or *GCKR* (Table 4). During follow‐up, 255 (46.7%) of the subjects in the NAFLD cohort died. No significant association was seen between any genotype or the PRS and higher risk of mortality. The same finding was seen in patients without advanced fibrosis at baseline (Table 5 and Figure 3).

**TABLE 4:** Hazard ratios from Cox regression analyses for associations between genotypes and cardiovascular disease events in the NAFLD cohort

Genotype	*N* exposed	*N* of outcomes (%)	Incidence rate (per 1000 person years)	HR (95% CI)	aHR^a^ (95% CI)
*PNPLA3* (C/C)	231	79 (34.2)	22.2 (17.8–27.7)	1.0 (ref)	1.0 (ref)
*PNPLA3* (C/G)	230	76 (33.0)	20.9 (16.7–26.1)	0.97 (0.71–1.31)	1.04 (0.76–1.44)
*PNPLA3* (G/G)	85	19 (22.3)	15.3 (9.8–24.0)	0.64 (0.39–1.05	0.64 (0.38–1.08)
*TM6SF2* (C/C)	386	124 (32.1)	21.2 (17.8–25.3)	1.0 (ref)	1.0 (ref)
*TM6SF2* (C/T)	116	37 (31.9)	20.1 (14.5–27.7)	0.85 (0.60–1.22)	0.75 (0.51–1.10)
*TM6SF2* (T/T)	21	8 (38.1)	19.4 (9.7–38.7)	0.87 (0.42–1.77)	1.17 (0.55–2.53)
*GCKR* (C/C)	209	69 (33.0)	20.2 (16.0–25.6)	1.0 (ref)	1.0 (ref)
*GCKR* (C/T)	219	64 (29.2)	19.2 (15.0–24.5)	0.97 (0.70–1.35)	0.99 (0.70–1.40)
*GCKR* (T/T)	107	36 (33.6)	23.2 (16.8–32.2)	1.24 (0.84–1.84)	1.19 (0.79–1.79)
*MBOAT7* (C/C)	187	64 (34.2)	22.6 (17.7–28.9)	1.0 (ref)	1.0 (ref)
*MBOAT7* (C/T)	234	70 (29.9)	18.8 (14.9–23.8)	0.82 (0.59–1.14)	0.84 (0.60–1.18)
*MBOAT7* (T/T)	111	37 (33.3)	22.6 (16.3–31.1)	1.00 (0.68–1.47)	1.07 (0.71–1.63)
PRS	507	175 (34.5)	—	0.68 (0.38–1.24)	0.73 (0.39–1.37)

*Note*: For PRS, the HR shows the association between PRS = 1 and the outcome compared with a PRS = 0 (ref.).

Abbreviations: aHR, adjusted hazard ratio; BMI, body mass index; CI, confidence interval; HR, hazard ratio, NAFLD, non‐alcoholic fatty liver disease; PRS, polygenic risk score.

^a^ Adjusted for age, sex, type 2 diabetes mellitus, BMI.

**TABLE 5:** Hazard ratios from Cox regression analyses for associations between genotypes and overall mortality in the NAFLD cohort

Genotype	*N* exposed	*N* of outcomes (%)	Incidence rate (per 1000 person years)	HR (95% CI)	aHR^a^ (95% CI)
*PNPLA3* (C/C)	231	101 (43.7)	23.6 (19.4–28.7)	1.0 (ref)	1.0 (ref)
*PNPLA3* (C/G)	230	102 (44.3)	23.6 (19.4–28.7)	1.03 (0.79–1.36)	0.93 (0.69–1.25)
*PNPLA3* (G/G)	85	32 (37.6)	22.8 (16.1–32.3)	1.03 (0.69–1.53)	1.14 (0.76–1.73)
*TM6SF2* (C/C)	386	173 (44.8)	24.8 (21.3–28.8)	1.0 (ref)	1.0 (ref)
*TM6SF2* (C/T)	116	44 (37.9)	20.5 (15.2–27.6)	0.75 (0.54–1.04)	0.77 (0.54–1.10)
*TM6SF2* (T/T)	21	10 (47.6)	21.7 (11.7–40.3)	0.84 (0.44–1.59)	1.33 (0.68–2.63)
*GCKR* (C/C)	209	101 (48.3)	25.4 (20.9–30.9)	1.0 (ref)	1.0 (ref)
*GCKR* (C/T)	219	82 (37.4)	21.0 (16.9–26.1)	0.76 (0.57–1.02)	0.80 (0.58–1.08)
*GCKR* (T/T)	107	46 (43.0)	23.8 (17.8–31.8)	0.90 (0.63–1.27)	0.80 (0.55–1.16)
*MBOAT7* (C/C)	187	86 (46.0)	25.8 (20.9–31.9)	1.0 (ref)	1.0 (ref)
*MBOAT7* (C/T)	234	95 (40.6)	21.2 (17.4–25.9)	0.86 (0.64–1.14)	0.87 (0.64–1.18)
*MBOAT7* (T/T)	111	47 (42.3)	24.2 (18.2–32.2)	0.88 (0.61–1.24)	0.74 (0.50–1.10)
PRS	392	190 (48.5)	—	0.78 (0.46–1.31)	0.84 (0.47–1.50)

*Note*: For PRS, the HR shows the association between PRS = 1 and the outcome compared with a PRS = 0 (ref.).

Abbreviations: aHR, adjusted hazard ratio; BMI, body mass index; CI, confidence interval; HR, hazard ratio; NAFLD, non‐alcoholic fatty liver disease; PRS, polygenic risk score.

^a^ Adjusted for age, sex, type 2 diabetes mellitus and BMI.

### 3.5 Association between allele type and rate of severe liver disease compared with the reference population and siblings

In total, 5234 controls were matched to the 546 NAFLD subjects with *PNPLA3* data. A total of 94 subjects in the reference population (1.8%) developed severe liver disease during follow‐up and 74 (13.5%) in the NAFLD cohort. Subjects with NAFLD exhibited in general a higher rate of severe liver disease compared with the reference population (HR 9.00, 95% CI = 6.85–11.83). The rate was further increased among carriers of the G/G‐genotype of *PNPLA3* (HR C/C vs controls: 6.79, 95% CI = 4.02–11.48; HR G/G vs controls: 23.32, 95% CI = 9.14–59.47; *p*_interaction_ = 0.017). For *TM6SF2*, *GCKR*, or *MBOAT7*, no association between genotype and rate of severe liver disease was observed (Table 6).

**TABLE 6:** Associations between genotype and development of severe liver disease in the NAFLD cohort compared with reference individuals matched for age, sex and municipality

Genotype	NAFLD cases (*n*)	Reference population (*n*)	Outcomes NAFLD	Outcomes Ref pop	Incidence rate, NAFLD (per 1000 person years)	Incidence rate, ref pop (per 1000 person years)	HR (95% CI)
*PNPLA3* (C/C)	231	2193	26	48	6.3 (4.3–9.2)	1.0 (0.7–1.3)	6.79 (4.02–11.48)
*PNPLA3* (C/G)	230	2225	31	34	7.5 (5.3–10.6)	0.7 (0.5–1.0)	11.77 (6.72–20.60)
*PNPLA3* (G/G)	85	816	17	12	13.2 (8.2–21.1)	0.7 (0.4–1.3)	23.32 (9.14–59.47)
*TM6SF2* (C/C)	386	3832	52	61	7.8 (5.9–10.2)	0.8 (0.6–1.0)	10.38 (6.87–15.67)
*TM6SF2* (C/T)	116	1138	15	26	7.2 (4.3–12.0)	1.1 (0.8–1.6)	9.50 (4.39–20.57)
*TM6SF2* (T/T)	21	209	3	4	6.7 (2.1–20.7)	0.9 (0.3–2.5)	7.63 (1.51–39.67)
*GCKR* (C/C)	209	2074	31	39	8.2 (5.7–11.6)	0.8 (0.7–1.2)	8.22 (4.98–13.56)
*GCKR* (C/T)	219	2166	29	40	7.7 (5.4–11.1)	0.9 (0.7–1.3)	11.22 (6.30–19.97)
*GCKR* (T/T)	107	1059	14	14	7.5 (4.4–12.7)	0.7 (0.4–1.1)	16.82 (6.76–41.86)
*MBOAT7* (C/C)	187	1847	33	35	10.4 (7.4–14.7)	1.0 (0.7–1.3)	11.86 (6.67–19.53)
*MBOAT7* (C/T)	234	2325	25	40	5.8 (3.9–8.6)	0.8 (0.6–1.1)	7.43 (4.29–12.89)
*MBOAT7* (T/T)	111	1098	14	17	7.4 (4.4–12.6)	0.8 (0.5–1.2)	14.79 (6.18–35.38)

Abbreviations: BMI, body mass index; CI, confidence interval; HR, hazard ratio; NAFLD, non‐alcoholic fatty liver disease; Ref pop, reference population.

Severe liver disease among siblings was rare (*n* = 18) and, therefore, no meaningful analyses could be performed.

### 3.6 Association between allele type, mortality and CVD in NAFLD compared with the reference population and siblings

Subjects with NAFLD had a higher rate of death compared with matched controls (HR 1.33, 95% CI = 1.17–1.50) and siblings (HR 1.68, 95% CI = 1.17–2.42). However, no further increased rate of mortality was observed between any allele type of the four genes in the NAFLD cohort compared with the reference population (Table S2) or siblings (Table S3).

NAFLD was associated with a higher rate of CVD compared with matched controls (HR 1.60, 95% CI = 1.39–1.86). No statistical difference in this rate was observed between any genotype of the *PNPLA3*, *TM6SF2*, *MBOAT7* or *GCKR* (Table S4). Data on events of CVD in siblings were not available because of restrictions in data access from the national registers, why no analysis was possible.

## 4 DISCUSSION

In this large cohort study, we found that the *PNPLA3* G/G genotype was associated with a more than twofold rate for the development of cirrhosis in NAFLD. The finding was consistent, both compared with other patients with NAFLD that carried the C/C genotype but also compared with reference individuals from the general population. Our results support a link between the G/G genotype in *PNPLA3* and risk for the development of both cirrhosis and HCC in NAFLD. However, the association was restricted to patients without advanced fibrosis at baseline. This demonstrates that to establish the *PNPLA3* genotype to estimate the risk of future liver‐related events, is of interest mainly for patients who are being diagnosed with NAFLD early after onset. For patients who already have developed advanced fibrosis at diagnosis, the disease promoting effect of the G/G genotype seems to be of less importance.

The results are consistent with previous findings. In a recent study on more than 80 000 obese individuals from the UK biobank with similar outcome measures, the *PNPLA3* C > G allele was shown to increase the risk of severe liver disease 1.6‐fold.^37^ In addition, in a recent study on 471 NAFLD patients prospectively enrolled and followed for a median of 5.4 years, a twofold increased risk of severe liver disease among carriers of the C > G allele of *PNPLA3* was reported.^38^ Our data extend these findings by a much longer follow‐up and by active comparison with the general population.

The effect of the *PNPLA3* G/G genotype on the risk of developing severe liver disease was more pronounced for patients without advanced fibrosis at baseline. Fibrosis is the most important predictor of long‐term prognosis in NAFLD.^26^ A plausible reason could be that in patients who have already developed advanced fibrosis, the additive effect on progression caused by genetic traits is of lower importance. Another explanation could be that the subgroup with advanced fibrosis was small and that the analysis was underpowered.

The *PNPLA3* G/G genotype was also associated with the presence of NASH at baseline. Our results are consistent with previous evidence supporting this relationship.^39^ However, we found no association between *PNPLA3* genotype and advanced fibrosis at baseline. This contrasts several previous studies that links *PNPLA3* genotype to advanced fibrosis.^40^, ^41^ This lack of association could possibly be the same as in our survival analysis, a low statistical power. Only 14.5% of histologically characterized subjects with NAFLD in our cohort had fibrosis stage 3 or 4 at baseline, compared with 35% in a previous study with a similar setting.^42^ This however also highlights that our cohort is less likely to suffer from selection bias.

We found that the PRS correlated to the presence of NASH but not to advanced fibrosis. This is partly consistent with the original study that first demonstrated that the PRS was predictive of both NASH and advanced fibrosis.^21^ In our study, the PRS did not correlate to increased risk of development of severe liver disease. We believe this discrepancy between previous reports and the present results is mainly explained by a lack of statistical power. The weight of each genotype in the original model that defines the PRS was based on the association to steatosis for each gene.^21^ The model was developed in a cohort of >9000 individuals. Only a minority of NAFLD patients with simple steatosis develop advanced liver disease during their lifetime. Therefore, our cohort of 546 genotyped NAFLD subjects was probably too small to gain enough statistical power to detect genetic variants with lesser effects on the long‐term prognosis in steatosis.

None of the *PNPLA3*, *TM6SF2*, *MBOAT7* or *GCKR* genotypes nor the PRS were associated with increased mortality. In a population‐based study of more than 19 000 individuals from the US National Health and Nutrition Examination Survey, the C > G allele of *PNPLA3* was associated with a 1.3 times risk of overall mortality and a 20‐fold risk of liver‐specific death.^22^ However, ours and other studies based on well‐defined NAFLD cohorts have not been able to verify these results.^38^ Unlike some previous results, our study did not find any significant associations between *TM6SF2*, *GCKR*, or *MBOAT7* and NAFLD severity.

Since the first reports describing *TM6SF2* and its suspected role in disease progression of NAFLD,^12^, ^43^ other studies have questioned this association. In a Japanese study, *TM6SF2* genotype was not associated with histological severity.^42^ In a study on the subjects with histologically characterized NAFLD and healthy controls, the *TM6SF2* was associated with a higher prevalence of NAFLD but not with liver fibrosis or NASH.^44^ Unlike previous reports, we found no association between mutations in *TM6SF2* and a reduced risk for CVD events.^45^ Although the evidence for an association between the *TM6SF2* gene and hepatic steatosis is robust, the disease promoting effect regarding long‐term development of liver cirrhosis or HCC remains unclear. The present study was not able to further establish such a correlation.

The *GCKR rs1260326* variant causes a loss‐of‐function of a regulatory enzyme that leads to increased de novo hepatic lipogenesis, thus increasing the risk of steatosis. However, simultaneous increased activity of intracellular glucokinase also leads to lower insulin resistance. Insulin resistance is itself a main driver of both NAFLD development and disease progression, and exactly how this dual mechanism affects NAFLD pathophysiology is not completely known.^46^, ^47^ The evidence for the importance of the *MBOAT7* gene is also diverging. Although an association with an increased risk of liver steatosis compared with non‐carriers can be established, recent studies have not been able to replicate the evidence for the impact of *MBOAT7* polymorphisms on disease severity in NAFLD.^48^, ^49^ In summary, the evidence for a link between the *GCKR* and *MBOAT7* genes and advanced histological disease or long‐term liver‐related events in NAFLD is lacking. Our results could not further strengthen such a correlation.

Our study has several strengths. The cohort consists of well characterized NAFLD subjects of which liver biopsy was available for the majority (91%). The identification of a matched reference population ensures a reliable comparison of outcomes with the general population. The follow‐up time of up to 40 years ensures that liver‐related outcomes could be detected even in a slowly progressing liver disease such as NAFLD.^50^ The use of nation‐wide registers leads to minimal loss to follow‐up. The ICD codes used to define severe liver disease in our study has recently been validated and found to be highly accurate.^51^

Some limitations should be acknowledged. The NAFLD cohort consisted of patients recruited from a clinical setting where they had been diagnosed with NAFLD owing to clinical symptoms or findings. Hence, there is a risk of selection bias, which could mean that the NAFLD cases in our cohort had more advanced liver disease compared with people with undiagnosed NAFLD found in the general population. This entails that a disease promoting gene, such as the *PNPLA3* G/G genotype, could be overrepresented in our material and would affect the external validity of our results. This is also inferred by the fact that 16% of the NAFLD cohort carried the *PNPLA3* G/G genotype compared with approximately 5% previously reported in studies of the general population.^52^

The cohort with available DNA data was limited to 546 subjects, which could mean that the study was underpowered to study rare outcomes such as severe liver disease for these genetic variants. However, this implies that the effect size of such associations might be limited and of little clinical relevance. There were no detailed data on the reference individuals or siblings. Still, the comparison is not possible to make outside of register‐based cohorts and can be a valuable addition to the field. Registry data can be less accurate in detecting alcohol use disorder since they are not always formally diagnosed. Therefore, there is a risk of misclassification bias in that alcohol use could be less frequently identified in the reference population that were not under active clinical care. This could lead to an imbalance between groups. However, such a bias would dilute the effect size towards the null and would not lead to false‐positive results. Living subjects were able to decline to participate in the study which was not the case for deceased subjects. This method for inclusion could lead to an over‐representation of deceased individuals and a selection bias. In 34 subjects, transient elastography was used to categorize fibrosis. This non‐invasive method is well established for clinical use but is not as accurate as liver biopsy, which is considered gold standard for detecting and grading of fibrosis.^53^ However, the prospective value of biopsy and elastography is similar.^54^

Our method for analysing DNA in tissue from liver biopsies of deceased subjects did not follow the same protocol as for living subjects, whose DNA was collected by blood tests. Any differences in accuracy, due to contamination or other complications between the two methods cannot be ruled out.

Moreover, the study was planned and initiated before discovery of other potentially interesting SNP:s such as in the *HSD17B13* gene.^55^ Future studies are needed to examine the long‐term effect of such genes on outcomes.

## 5 CONCLUSION

The G/G genotype (I148M) of the *PNPLA3* gene was associated with a higher rate of progression to severe liver disease in patients with NAFLD and particularly in patients without advanced fibrosis at diagnosis. Genotyping of the *PNPLA3* gene might be of clinical importance when tailoring future surveillance programs for patients with NAFLD.

## ETHICAL APPROVAL

The study was approved by the regional ethics committee in Stockholm, Sweden. (Dnr 2011/905–31/2, 2011/13–31/1, 2016/2137–31, 2018/880–31). All subjects included in the NAFLD cohort that were alive at the time of inclusion signed an informed consent.

## FUNDING INFORMATION

HH was supported by grants from Region Stockholm and the Swedish Cancer Society.

## CONFLICT OF INTEREST

The authors do not have any disclosures to report.

## Supporting information


Appendix S1

## References

[1] Younossi ZM , Koenig AB , Abdelatif D , Fazel Y , Henry L , Wymer M . Global epidemiology of nonalcoholic fatty liver disease‐Meta‐analytic assessment of prevalence, incidence, and outcomes. Hepatology. 2016;64(1):73‐84. doi:10.1002/hep.28431 26707365

[2] Younossi ZM , Golabi P , de Avila L , et al. The global epidemiology of NAFLD and NASH in patients with type 2 diabetes: a systematic review and meta‐analysis. J Hepatol. 2019;71(4):793‐801. doi:10.1016/j.jhep.2019.06.021 31279902

[3] EASL‐EASD‐EASO Clinical Practice Guidelines for the management of non‐alcoholic fatty liver disease. J Hepatol. 2016;64(6):1388‐1402. doi:10.1016/j.jhep.2015.11.004 27062661

[4] McPherson S , Hardy T , Henderson E , Burt AD , Day CP , Anstee QM . Evidence of NAFLD progression from steatosis to fibrosing‐steatohepatitis using paired biopsies: implications for prognosis and clinical management. J Hepatol. 2015;62(5):1148‐1155. doi:10.1016/j.jhep.2014.11.034 25477264

[5] Loomba R , Schork N , Chen CH , et al. Heritability of hepatic fibrosis and steatosis based on a prospective twin study. Gastroenterology. 2015;149(7):1784‐1793. doi:10.1053/j.gastro.2015.08.011 26299412 PMC4663110

[6] Struben VM , Hespenheide EE , Caldwell SH . Nonalcoholic steatohepatitis and cryptogenic cirrhosis within kindreds. Am J Med. 2000;108(1):9‐13. doi:10.1016/s0002-9343(99)00315-0 11059435

[7] Dongiovanni P , Anstee QM , Valenti L . Genetic predisposition in NAFLD and NASH: impact on severity of liver disease and response to treatment. Curr Pharm Des. 2013;19(29):5219‐5238. doi:10.2174/13816128113199990381 23394097 PMC3850262

[8] Romeo S , Kozlitina J , Xing C , et al. Genetic variation in PNPLA3 confers susceptibility to nonalcoholic fatty liver disease. Nat Genet. 2008;40(12):1461‐1465. doi:10.1038/ng.257 18820647 PMC2597056

[9] Huang Y , Cohen JC , Hobbs HH . Expression and characterization of a PNPLA3 protein isoform (I148M) associated with nonalcoholic fatty liver disease. J Biol Chem. 2011;286(43):37085‐37093. doi:10.1074/jbc.M111.290114 21878620 PMC3199456

[10] Pingitore P , Romeo S . The role of PNPLA3 in health and disease. Biochim Biophys Acta Mol Cell Biol Lipids. 2019;1864(6):900‐906. doi:10.1016/j.bbalip.2018.06.018 29935383

[11] Sookoian S , Pirola CJ . Meta‐analysis of the influence of I148M variant of patatin‐like phospholipase domain containing 3 gene (PNPLA3) on the susceptibility and histological severity of nonalcoholic fatty liver disease. Hepatology. 2011;53(6):1883‐1894. doi:10.1002/hep.24283 21381068

[12] Liu YL , Reeves HL , Burt AD , et al. TM6SF2 rs58542926 influences hepatic fibrosis progression in patients with non‐alcoholic fatty liver disease. Nat Commun. 2014;5:4309. doi:10.1038/ncomms5309 24978903 PMC4279183

[13] Luukkonen PK , Zhou Y , Hyotylainen T , et al. The MBOAT7 variant rs641738 alters hepatic phosphatidylinositols and increases severity of non‐alcoholic fatty liver disease in humans. J Hepatol. 2016;65(6):1263‐1265. doi:10.1016/j.jhep.2016.07.045 27520876

[14] Mancina RM , Dongiovanni P , Petta S , et al. The MBOAT7‐TMC4 Variant rs641738 increases risk of nonalcoholic fatty liver disease in individuals of European descent. Gastroenterology. 2016;150(5):1219‐1230.e6. doi:10.1053/j.gastro.2016.01.032 26850495 PMC4844071

[15] Umano GR , Caprio S , Di Sessa A , et al. The rs626283 variant in the MBOAT7 gene is associated with insulin resistance and fatty liver in Caucasian obese youth. Am J Gastroenterol. 2018;113(3):376‐383. doi:10.1038/ajg.2018.1 29485130 PMC12136689

[16] Beer NL , Tribble ND , McCulloch LJ , et al. The P446L variant in GCKR associated with fasting plasma glucose and triglyceride levels exerts its effect through increased glucokinase activity in liver. Human Mol Genet. 2009;18(21):4081‐4088. doi:10.1093/hmg/ddp357 19643913 PMC2758140

[17] Speliotes EK , Yerges‐Armstrong LM , Wu J , et al. Genome‐wide association analysis identifies variants associated with nonalcoholic fatty liver disease that have distinct effects on metabolic traits. PLoS Genet. 2011;7(3):e1001324. doi:10.1371/journal.pgen.1001324 21423719 PMC3053321

[18] Santoro N , Zhang CK , Zhao H , et al. Variant in the glucokinase regulatory protein (GCKR) gene is associated with fatty liver in obese children and adolescents. Hepatology. 2012;55(3):781‐789. doi:10.1002/hep.24806 22105854 PMC3288435

[19] Petit JM , Masson D , Guiu B , et al. GCKR polymorphism influences liver fat content in patients with type 2 diabetes. Acta Diabetol. 2016;53(2):237‐242. doi:10.1007/s00592-015-0766-4 25976242

[20] Petta S , Miele L , Bugianesi E , et al. Glucokinase regulatory protein gene polymorphism affects liver fibrosis in non‐alcoholic fatty liver disease. PloS One. 2014;9(2):e87523. doi:10.1371/journal.pone.0087523 24498332 PMC3911959

[21] Dongiovanni P , Stender S , Pietrelli A , et al. Causal relationship of hepatic fat with liver damage and insulin resistance in nonalcoholic fatty liver. J Intern Med. 2018;283(4):356‐370. doi:10.1111/joim.12719 29280273 PMC5900872

[22] Unalp‐Arida A , Ruhl CE . Patatin‐like phospholipase domain‐containing protein 3 I148M and liver fat and fibrosis scores predict liver disease mortality in the U.S. population. Hepatology. 2020;71(3):820‐834. doi:10.1002/hep.31032 31705824

[23] Walker RW , Belbin GM , Sorokin EP , et al. A common variant in PNPLA3 is associated with age at diagnosis of NAFLD in patients from a multi‐ethnic biobank. J Hepatol. 2020;72(6):1070‐1081. doi:10.1016/j.jhep.2020.01.029 32145261 PMC7840172

[24] Oniki K , Saruwatari J , Izuka T , et al. Influence of the PNPLA3 rs738409 polymorphism on non‐alcoholic fatty liver disease and renal function among normal weight subjects. PloS ONE. 2015;10(7):e0132640. doi:10.1371/journal.pone.0132640 26200108 PMC4511733

[25] Hagström H , Nasr P , Ekstedt M , et al. SAF score and mortality in NAFLD after up to 41 years of follow‐up. Scand J Gastroenterol. 2017;52(1):87‐91. doi:10.1080/00365521.2016.1230779 27616339

[26] Hagstrom H , Nasr P , Ekstedt M , et al. Fibrosis stage but not NASH predicts mortality and time to development of severe liver disease in biopsy‐proven NAFLD. J Hepatol. 2017;67(6):1265‐1273. doi:10.1016/j.jhep.2017.07.027 28803953

[27] Laugesen K , Ludvigsson JF , Schmidt M , et al. Nordic health registry‐based research: A review of health care systems and key registries. Clin Epidemiol. 2021;13:533‐554. doi:10.2147/clep.S314959 34321928 PMC8302231

[28] Kleiner DE , Brunt EM , Van Natta M , et al. Design and validation of a histological scoring system for nonalcoholic fatty liver disease. Hepatology. 2005;41(6):1313‐1321. doi:10.1002/hep.20701 15915461

[29] Bedossa P . Utility and appropriateness of the fatty liver inhibition of progression (FLIP) algorithm and steatosis, activity, and fibrosis (SAF) score in the evaluation of biopsies of nonalcoholic fatty liver disease. Hepatology. 2014;60(2):565‐575. doi:10.1002/hep.27173 24753132

[30] Raja AM , Ciociola E , Ahmad IN , et al. Genetic susceptibility to chronic liver disease in individuals from Pakistan. Int J Mol Sci. 2020;21(10). doi:10.3390/ijms21103558 PMC7278956 32443539

[31] Holland PM , Abramson RD , Watson R , Gelfand DH . Detection of specific polymerase chain reaction product by utilizing the 5′‐3′ exonuclease activity of Thermus aquaticus DNA polymerase. Proc Natl Acad Sci USA. 1991;88(16):7276‐7280. doi:10.1073/pnas.88.16.7276 1871133 PMC52277

[32] Ludvigsson JF , Andersson E , Ekbom A , et al. External review and validation of the Swedish national inpatient register. BMC Public Health. 2011;11:450. doi:10.1186/1471-2458-11-450 21658213 PMC3142234

[33] Ludvigsson JF , Otterblad‐Olausson P , Pettersson BU , Ekbom A . The Swedish personal identity number: possibilities and pitfalls in healthcare and medical research. Eur J Epidemiol. 2009;24(11):659‐667. doi:10.1007/s10654-009-9350-y 19504049 PMC2773709

[34] Barlow L , Westergren K , Holmberg L , Talbäck M . The completeness of the Swedish Cancer Register: a sample survey for year 1998. Acta Oncol. 2009;48(1):27‐33. doi:10.1080/02841860802247664 18767000

[35] Brooke HL , Talbäck M , Hörnblad J , et al. The Swedish cause of death register. Eur J Epidemiol. 2017;32(9):765‐773. doi:10.1007/s10654-017-0316-1 28983736 PMC5662659

[36] Austin PC , Lee DS , Fine JP . Introduction to the analysis of survival data in the presence of competing risks. Circulation. 2016;133(6):601‐609. doi:10.1161/circulationaha.115.017719 26858290 PMC4741409

[37] De Vincentis A , Tavaglione F , Spagnuolo R , et al. Metabolic and genetic determinants for progression to severe liver disease in subjects with obesity from the UK Biobank. Int J Obes. 2021;46:486‐493. doi:10.1038/s41366-021-01015-w PMC8573310 34750514

[38] Grimaudo S , Pipitone RM , Pennisi G , et al. Association between PNPLA3 rs738409 C>G variant and liver‐related outcomes in patients with nonalcoholic fatty liver disease. Clin Gastroenterol Hepatol. 2020;18(4):935‐944.e3. doi:10.1016/j.cgh.2019.08.011 31419571

[39] Kozlitina J . Genetic risk factors and disease modifiers of nonalcoholic steatohepatitis. Gastroenterol Clin North Am. 2020;49(1):25‐44. doi:10.1016/j.gtc.2019.09.001 32033763

[40] Valenti L , Al‐Serri A , Daly AK , et al. Homozygosity for the patatin‐like phospholipase‐3/adiponutrin I148M polymorphism influences liver fibrosis in patients with nonalcoholic fatty liver disease. Hepatology. 2010;51(4):1209‐1217. doi:10.1002/hep.23622 20373368

[41] Pennisi G , Pipitone RM , Cammà C , et al. PNPLA3 rs738409 C>G variant predicts fibrosis progression by noninvasive tools in nonalcoholic fatty liver disease. Clin Gastroenterol Hepatol. 2021;19(9):1979‐1981. doi:10.1016/j.cgh.2020.09.009 32898706

[42] Akuta N , Kawamura Y , Arase Y , et al. Relationships between genetic variations of PNPLA3, TM6SF2 and histological features of nonalcoholic fatty liver disease in Japan. Gut Liver. 2016;10(3):437‐445. doi:10.5009/gnl15163 26610348 PMC4849698

[43] Kozlitina J , Smagris E , Stender S , et al. Exome‐wide association study identifies a TM6SF2 variant that confers susceptibility to nonalcoholic fatty liver disease. Nat Genet. 2014;46(4):352‐356. doi:10.1038/ng.2901 24531328 PMC3969786

[44] Sookoian S , Castaño GO , Scian R , et al. Genetic variation in transmembrane 6 superfamily member 2 and the risk of nonalcoholic fatty liver disease and histological disease severity. Hepatology. 2015;61(2):515‐525. doi:10.1002/hep.27556 25302781

[45] Dongiovanni P , Petta S , Maglio C , et al. Transmembrane 6 superfamily member 2 gene variant disentangles nonalcoholic steatohepatitis from cardiovascular disease. Hepatology. 2015;61(2):506‐514. doi:10.1002/hep.27490 25251399

[46] Bugianesi E , Gastaldelli A , Vanni E , et al. Insulin resistance in non‐diabetic patients with non‐alcoholic fatty liver disease: sites and mechanisms. Diabetologia 2005;48(4):634–42. doi:10.1007/s00125-005-1682-x 15747110

[47] Brouwers M , Jacobs C , Bast A , Stehouwer CDA , Schaper NC . Modulation of glucokinase regulatory protein: a double‐edged sword? Trends Mol Med. 2015;21(10):583‐594. doi:10.1016/j.molmed.2015.08.004 26432016

[48] Sookoian S , Flichman D , Garaycoechea ME , et al. Lack of evidence supporting a role of TMC4‐rs641738 missense variant‐MBOAT7‐intergenic downstream variant‐in the Susceptibility to Nonalcoholic Fatty Liver Disease. Scient Rep. 2018;8(1):5097. doi:10.1038/s41598-018-23453-9 PMC5865142 29572551

[49] Anstee QM , Darlay R , Cockell S , et al. Genome‐wide association study of non‐alcoholic fatty liver and steatohepatitis in a histologically characterised cohort(☆). J Hepatol. 2020;73(3):505‐515. doi:10.1016/j.jhep.2020.04.003 32298765

[50] Singh S , Allen AM , Wang Z , Prokop LJ , Murad MH , Loomba R . Fibrosis progression in nonalcoholic fatty liver vs nonalcoholic steatohepatitis: a systematic review and meta‐analysis of paired‐biopsy studies. Clin Gastroenterol Hepatol. 2015;13(4):643‐654.e1‐9; quiz e39‐40. doi:10.1016/j.cgh.2014.04.014 24768810 PMC4208976

[51] Bengtsson B , Askling J , Ludvigsson JF , Hagström H . Validity of administrative codes associated with cirrhosis in Sweden. Scand J Gastroenterol. 2020;55(10):1205‐1210. doi:10.1080/00365521.2020.1820566 32960654

[52] Severson TJ , Besur S , Bonkovsky HL . Genetic factors that affect nonalcoholic fatty liver disease: A systematic clinical review. World J Gastroenterol. 2016;22(29):6742‐6756. doi:10.3748/wjg.v22.i29.6742 27547017 PMC4970479

[53] de Franchis R . Expanding consensus in portal hypertension: Report of the Baveno VI Consensus Workshop: Stratifying risk and individualizing care for portal hypertension. J Hepatol. 2015;63(3):743‐752. doi:10.1016/j.jhep.2015.05.022 26047908

[54] Boursier J , Hagström H , Ekstedt M , et al. Non‐invasive tests accurately stratify patients with NAFLD based on their risk of liver‐related events. J Hepatology. 2022;76(5):1013‐1020. doi:10.1016/j.jhep.2021.12.031 35063601

[55] Ma Y , Belyaeva OV , Brown PM , et al. 17‐beta hydroxysteroid dehydrogenase 13 is a hepatic retinol dehydrogenase associated with histological features of nonalcoholic fatty liver disease. Hepatology. 2019;69(4):1504‐1519. doi:10.1002/hep.30350 30415504 PMC6438737

