# Supplementary material for: Effect of common genetic variants on the risk of cirrhosis in non‐alcoholic fatty liver disease during 20 years of follow‐up
Source: Liver Int. 2022 Oct 11;42(12):2769–80. doi: 10.1111/liv.15438 (PMC9828463; doi:10.1111/liv.15438)
Supplement: Supplementary file 1 — Appendix S1 [file LIV-42-2769-s001.docx]

**Supplementary table 1.** ICD-codes defining exclusion criteria and liver-related outcomes

| **Diagnosis** | **ICD-8** | **ICD-9** | **ICD-10** |
| --- | --- | --- | --- |
| **Excluding NAFLD at or before baseline, ONLY done in reference population** | | | |
| NAFLD, all | No available code | 571.8 | K76.0 |
| NASH | No available code | No available code | K75.8 |
| **Excluding other liver diseases at/before baseline in reference population** | | | |
| ALD | 571,00, 571,01 | 571.0-571.3 | K70 |
| Viral hepatitis | 999,2, 070 | 070 | B16, B17, B18, B19 |
| Autoimmune liver disease (AIH, PBC, PSC) | No available code | 571.6, 576.1 | K83.0A, K83.0F, K74.3, K75.4 |
| Hemochromatosis | 273,2 | 275.0 | E83.1 |
| Wilson | 273,3 | 275.1 | E83.0B |
| Alpha-1-antitrypsin deficiency | No available code | 277.6 | E88.0A, E88.0B |
| Budd-Chiari | No available code | 453.0 | I82.0, K76.5 |
| Chronic hepatitis, unspecified | 570 | 571.4 | K73.9, K73.2 |
| Secondary or unspecified biliary cirrhosis | No available code | 571.6 | K74.4, K74.5 |
| **Excluding alcohol/drug use disorder at/before baseline in reference population** | | | |
| Codes associated with alcohol use disorder | 303 | 303, 305.0 | F10 |
| Codes associated with somatic consequences of alcohol (except ALD) | 291, 980,00, 980,01, 980,99 | 291, 357.5, 425.5, 535.3, 980.1, 980.9 | E24.4, G62.1, I42.6, K29.2, G31.2, G72.1, K85.2, K86.0, T51.0, T51.9, Y57.3, X65, Z50.2, Z71.4, Z72.1 |
| Codes associated with drug use disorders except nicotine/caffeine | No available code | 305.1-9 | F11-F14, F16, F18, F19 |
| **Compensated cirrhosis outcomes (included in severe liver disease definition)** | | | |
| Cirrhosis, compensated | 571,9 | 571.5 | K74.6 |
| Esophageal varices, not bleeding | No available code | 456.1, 456.21 | I85.9, I98.2 |
| Gastric varices, not bleeding | No available code | No available code | I86.4 |
| **Decompensated cirrhosis outcomes (included in severe liver disease definition)** | | | |
| Esophageal varices, bleeding | 456.0 | 456.0, 456.20 | I85.0, I98.3 |
| Ascites | 785.3 | 789.5 | R18 |
| Hepatic encephalopathy | No available code | 572.2 | No available code |
| Hepatorenal syndrome | No available code | 572.4 | K76.7 |
| Portal hypertension | 571,9 | 572.3 | K76.6 |
| **Liver transplantation outcomes (included in severe liver disease definition)** | | | |
| Liver transplantation status | No available code | V427 | Z94.4 |
| **Liver cancer outcomes (included in severe liver disease definition)** | | | |
| HCC | 155,01 | 155.0 | C22.0 |
| Liver cancer, unspecified | No available code | 155.2 | C22.9 |
| **Cardiovascular disease outcome** | | | |
| Cerebrovascular disease | 431, 432 | 431, 433 | I61, I63, I64 |
| Acute ischemic heart disease | 410-413 | 410-413 | I20-I24 |

Abbreviations: ICD= international classification of diseases, NAFLD= nonalcoholic fatty liver disease, NASH= nonalcoholic steatohepatitis, ALD= Alcoholic liver disease, AIH= autoimmune hepatitis, PBC= primary sclerosing cholangitis, PBC, primary biliary cholangitis, HCC= hepatocellular carcinoma

| **Genotype** | **NAFLD cases (n)** | **Reference population (n)** | **Outcomes NAFLD** | **Outcomes, ref pop** | **Incidence rate, NAFLD (per 1000 person years)** | **Incidence rate, ref pop (per 1000 person years)** | **HR**  **(95%CI)** |
| --- | --- | --- | --- | --- | --- | --- | --- |
| PNPLA3 (C/C) | 231 | 2227 | 101 | 843 | 23.6 (19.4-28.7) | 17.8 (16.6-19.0) | 1.52 (1.22-1.90) |
| PNPLA3 (C/G) | 230 | 2243 | 102 | 786 | 23.6 (19.5-28.7) | 16.7 (15.6-18.0) | 1.38 (1.11-1.72) |
| PNPLA3 (G/G) | 85 | 823 | 32 | 258 | 22.8 (16.1-32.3) | 16.0 (14.1-18.0) | 1.65 (1.11-2.44) |
| TM6SF2 (C/C) | 386 | 3748 | 173 | 1356 | 24.8 (21.4-28.8) | 17.4 (16.6-18.4) | 1.49 (1.26-1.77) |
| TM6SF2 (C/T) | 116 | 1118 | 44 | 391 | 20.5 (15.3-27.6) | 16.1 (14.6-17.8) | 1.41 (1.01-1.96) |
| TM6SF2 (T/T) | 21 | 203 | 10 | 71 | 21.7 (11.7-40.3) | 16.6 (13.1-21.0) | 1.51 (0.75-3.02) |
| GCKR (C/C) | 209 | 2034 | 101 | 763 | 25.4 (20.9-30.9) | 17.0 (15.9-18.3) | 1.71 (1.37-2.14) |
| GCKR (C/T) | 219 | 2111 | 82 | 706 | 21.0 (16.9-26.1) | 16.4 (15.2-17.7) | 1.31 (1.03-1.68) |
| GCKR (T/T) | 107 | 1040 | 46 | 369 | 23.8 (17.8-31.8) | 18.1 (16.3-20.0) | 1.36 (0.98-1.89) |
| MBOAT7 (C/C) | 187 | 1800 | 86 | 648 | 25.8 (20.9-31.9) | 17.7 (16.4-19.1) | 1.62 (1.27-2.06) |
| MBOAT7 (C/T) | 234 | 2278 | 95 | 797 | 21.2 (17.4-25.9) | 16.4 (15.3-17.5) | 1.38 (1.10-1.73) |
| MBOAT7 (T/T) | 111 | 1078 | 47 | 381 | 24.2 (18.2-32.2) | 17.2 (15.6-19.0) | 1.39 (1.01-1.93) |

**Supplementary table 2**. Associations between genotype and overall mortality in the NAFLD cohort compared with reference individuals matched for age, sex and municipality. Abbreviations: CI= confidence interval, NAFLD= nonalcoholic fatty liver disease, HR= hazard ratio, Ref pop= reference population.

| **Genotype** | **NAFLD cases (n)** | **First-degree relatives (n)** | **Outcomes NAFLD** | **Outcomes, first degree relatives** | **Incidence rate, NAFLD (per 1000 person years)** | **Incidence rate, first-degree relatives (per 1000 person years)** | **HR**  **(95%CI)** |
| --- | --- | --- | --- | --- | --- | --- | --- |
| PNPLA3 (C/C) | 89 | 160 | 17 | 21 | 10.8 (6.7-17.4) | 5.6 (3.7-8.7) | 3.16 (1.42-7.02) |
| PNPLA3 (C/G) | 85 | 187 | 23 | 37 | 14.0 (9.3-21.0) | 8.2 (5.9-11.3) | 2.17 (1.19-4.00) |
| PNPLA3 (G/G) | 33 | 70 | 5 | 15 | 9.6 (4.0-23.0) | 11.2 (6.7-18.6) | 1.09 (0.31-3.86) |
| TM6SF2 (C/C) | 139 | 269 | 32 | 49 | 12.8 (0.9-18.2) | 8.1 (6.1-10.7) | 2.09 (1.23-3.55) |
| TM6SF2 (C/T) | 51 | 115 | 9 | 20 | 10.1 (5.3-19.5) | 7.1 (4.6-11.1) | 2.31 (0.88-6.09) |
| TM6SF2 (T/T) | 8 | 16 | 2 | 1 | 9.2 (2.3-36.7) | 2.5 (0.3-17.8) | 3.00 (0.25-35.79) |
| GCKR (C/C) | 76 | 155 | 23 | 26 | 14.6 (9.7-22.0) | 7.0 (4.8-10.3) | 2.62 (1.35-5.10) |
| GCKR (C/T) | 87 | 171 | 14 | 29 | 10.1 (6.0-17.2) | 10.8 (5.4-11.2) | 2.25 (1.01-5.01) |
| GCKR (T/T) | 43 | 89 | 8 | 17 | 10.1 (5.1-20.3) | 8.1 (5.0-13.0) | 1.60 (0.62-4.14) |
| MBOAT7 (C/C) | 79 | 155 | 17 | 30 | 13.9 (8.6-22.3) | 8.9 (6.2-12.8) | 3.34 (1.55-7.20) |
| MBOAT7 (C/T) | 85 | 172 | 18 | 32 | 10.4 (6.6-16.5) | 8.1 (5.7-11.5) | 1.62 (0.85-3.09) |
| MBOAT7 (T/T) | 40 | 87 | 10 | 11 | 13.7 (7.3-25.5) | 5.0 (2.8-9.0) | 2.49 (0.85-7.32) |

**Supplementary table 3**. Associations between genotype and overall mortality in NAFLD patients compared with their first-degree relatives, adjusted for age, sex and municipality. Abbreviations: CI= confidence interval, NAFLD= nonalcoholic fatty liver disease, HR= hazard ratio, Ref pop= reference population.

| **Genotype** | **NAFLD cases (n)** | **Reference population (n)** | **Outcomes NAFLD** | **Outcomes, ref pop** | **Incidence rate, NAFLD (per 1000 person years)** | **Incidence rate, ref pop (per 1000 person years)** | **HR**  **(95%CI)** |
| --- | --- | --- | --- | --- | --- | --- | --- |
| PNPLA3 (C/C) | 231 | 2287 | 79 | 549 | 22.2 (17.8-27.7) | 12.6 (11.6-13.7) | 1.91 (1.48-2.46) |
| PNPLA3 (C/G) | 230 | 2286 | 76 | 543 | 20.9 (16.7-26.1) | 12.6 (11.6-13.7) | 1.77 (1.37-2.30) |
| PNPLA3 (G/G) | 85 | 836 | 19 | 197 | 15.3 (9.8-24.0) | 13.2 (11.5-15.2) | 1.38 (0.84-2.24) |
| TM6SF2 (C/C) | 386 | 3832 | 124 | 921 | 21.2 (17.8-25.3) | 12.9 (12.1-13.8) | 1.78 (1.45-2.17) |
| TM6SF2 (C/T) | 116 | 1138 | 37 | 269 | 20.1 (14.5-27.7) | 11.9 (10.6-13.5) | 1.82 (1.26-2.63) |
| TM6SF2 (T/T) | 21 | 209 | 8 | 50 | 19.4 (9.7-38.7) | 12.6 (9.6-16.7) | 1.46 (0.70-3.32) |
| GCKR (C/C) | 209 | 2074 | 69 | 523 | 20.2 (16.0-25.6) | 12.8 (11.7-13.9) | 1.81 (1.38-2.36) |
| GCKR (C/T) | 219 | 2166 | 64 | 477 | 19.2 (15.0-24.5) | 11.9 (10.9-13.1) | 1.67 (1.27-2.21) |
| GCKR (T/T) | 107 | 1059 | 36 | 251 | 23.2 (16.8-32.2) | 13.3 (11.8-15.1) | 1.95 (1.33-2.85) |
| MBOAT7 (C/C) | 187 | 1847 | 64 | 426 | 22.6 (17.7-28.9) | 12.6 (11.5-13.8) | 1.93 (1.45-2.57) |
| MBOAT7 (C/T) | 234 | 2325 | 70 | 566 | 18.8 (14.9-23.8) | 12.7 (11.7-13.8) | 1.65 (1.27-2.15) |
| MBOAT7 (T/T) | 111 | 1098 | 37 | 259 | 22.6 (16.3-31.1) | 12.6 (11.2-14.3) | 1.87 (1.29-2.71) |

**Supplementary table 4**. Associations between mutation genotype and risk of cardiovascular events in the NAFLD cohort compared with reference individuals matched for age, sex and municipality. Abbreviations: CI= confidence interval, NAFLD= nonalcoholic fatty liver disease, HR= hazard ratio, Ref pop= reference population.
